# High thermal conductivity driven by the unusual phonon relaxation time platform in 2D monolayer boron arsenide

**DOI:** 10.1039/d0ra04737f

**Published:** 2020-07-02

**Authors:** Yanxiao Hu, Dengfeng Li, Yan Yin, Shichang Li, Hangbo Zhou, Gang Zhang

**Affiliations:** School of Science, Chongqing University of Posts and Telecommunications Chongqing 400065 China lidf@cqupt.edu.cn; Department of Physics, Tsinghua University Beijing 100084 China; Institute of High Performance Computing, A*STAR 138632 Singapore zhangg@ihpc.a-star.edu.sg

## Abstract

The cubic boron arsenide (BAs) crystal has received extensive research attention because of its ultra-high thermal conductivity comparable to that of diamond. In this work, we performed a comprehensive study on the diffusive thermal properties of its two-dimensional (2D) counterpart, the monolayer honeycomb BAs (h-BAs), through solving the phonon Boltzmann transport equation combined with first-principles calculation. We found that unlike the pronounced contribution from out-of-plane acoustic phonons (ZA) in graphene, the high thermal conductivity (181 W m^−1^ K^−1^ at 300 K) of h-BAs is mainly contributed by in-plane phonon modes, instead of the ZA mode. This result is explained by the unique frequency-independent ‘platform’ region in the relaxation time of in-plane phonons. Moreover, we conducted a comparative study of thermal conductivity between 2D h-BAs and h-GaN, because both of them have a similar mass density. The thermal conductivity of h-BAs is one order of magnitude higher than that of h-GaN (16 W m^−1^ K^−1^), which is governed by the different phonon scattering process attributed to the opposite wavevector dependence in out-of-plane optical phonons. Our findings provide new insight into the physics of heat conduction in 2D materials, and demonstrate h-BAs to be a new thermally conductive 2D semiconductor.

## Abstract 2

The cubic boron arsenide (BAs) crystal has received extensive research attention because of its ultra-high thermal conductivity comparable to that of diamond.

## Graphical abstract

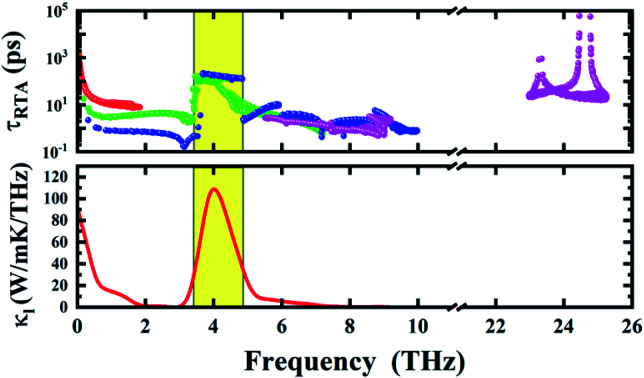

## 1. Introduction

With the rapid development of miniaturization and high-power density of the electronic and optoelectronic devices, the demand for thermal management is becoming more and more important.^1,2^ Diamond is a well-known highly thermally conductive bulk material. The decreasing dimension from three-dimensional diamond to two-dimensional (2D) graphene can change the phonon population distribution, and introduces emerging physical phenomena. For example, the high Debye temperature and the strong anharmonicity of flexural ZA (out-of-plane acoustic) phonon modes around the Γ point result in remarkable phonon hydrodynamics in graphene.^3,4^ Moreover, Lindsay *et al.*^5^ found a special selection rule in graphene, in which only three-phonon processes having an even number of ZA phonons are allowed, while other processes (involving one or three ZA phonons) are forbidden.^6,7^ This is one of the underlying mechanisms for the higher thermal conductivity in graphene exceeding diamond. The anomalous length-dependent thermal conductivity is also observed in graphene, revealing its promising perspective in thermal management.^8^

Inspired by the ultra-high thermal conductivity of graphene,^9^ the researches on thermal transport in low-dimensional system have attracted extensive attention.^10–16^ The decreased dimension induces quantum confinement effect and alters the phonon dispersion, which further results in unique lattice thermal transport features.^17^ In addition to graphene, Sahin *et al.*^18^ has proposed 13 different 2D III–V compounds of honeycomb lattice, seven of which (AlN, GaN, InN, BN, BP, BAs and BSb) have planer structure like graphene. In planer structures, phonons obey the unique phonon scattering selection rule^5,19,20^ in which three-phonon processes having an odd number of ZA phonons are forbidden. This mechanism can reduce phonon scattering rate by suppressing scattering channels, and consequently enhance thermal conductivity.

Within these III–V compounds of honeycomb lattice, the most famous one is honeycomb BN (h-BN), which exhibits good thermal transport property.^21–23^ Recently, it is demonstrated^24,25^ that monolayer honeycomb BAs (h-BAs) nanosheet is an excellent candidate for both electrically functional material and thermally functional material. For example, Zhang *et al.*^24^ reported the appropriate band gap (1.18 eV), high carrier mobility and strain tunable electronic property of h-BAs. Manoharan and Subramanian^25^ reported the *I*–*V* characteristics of h-BAs, and the potential application in short-wavelength optoelectronic devices was discussed. More interesting, recent theoretical^26–28^ and experimental^29–31^ works demonstrate high thermal conductivity in bulk BAs crystal (c-BAs), origins partially from the large frequency gap between acoustic and optical phonon modes. Naturally, it raises up some questions, for example, what is the thermal conductivity of its 2D counterpart (h-BAs)? Which phonon modes play dominant contribution to thermal conductivity? Compared with other 2D III–V honeycomb lattice, what is the unique phonon scheme in h-BAs? Although preliminary literatures reported high thermal conductivity of h-BAs,^32,33^ compared with the extensive studies of thermal conductivity of graphene and h-BN, there are still many open questions. Obviously, a better knowledge of thermal conductivity of h-BAs is highly important not only for fundamental physics of low-dimensional thermal transport, but also for technological applications such as thermal management of nanoscale integrated devices.

In this paper, based on first-principle calculation, the abnormal high thermal conductivity of h-BAs was revealed by solving phonon Boltzmann transport equation (BTE). We found the main contribution to thermal conductivity is from in-plane acoustic phonons and a frequency-independent ‘platform’ appearing in relaxation time of in-plane modes. By analyzing phonon dispersion and considering phonon selection rule, we explained our calculation results and found that phonon selection rule not only affects out-of-plane phonons but also plays an important role for in-plane phonons. Furthermore, the comparison of thermal transport of h-BAs with h-GaN highlights the effect of phonon spectrum on thermal transport.

## 2. Theoretical and computational methods

By solving phonon BTE, thermal conductivity tensor element of *αβ* direction at temperature *T* can be expressed as the sum of contributions from each phonon modes λ:1
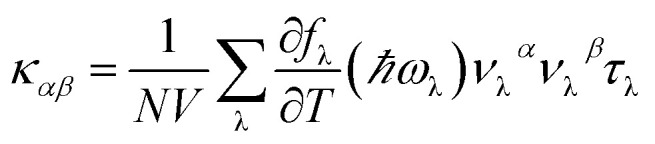
where *N*, *V*, *f*_λ_, *ν* and *τ* are the number of wave vector *q* points in the Brillouin zone, volume of the unit cell, the equilibrium Boltzmann distribution depending on phonon angular frequency *ω*_λ_, phonon group velocity and phonon relaxation time, respectively. Under relaxation time approximation (RTA), phonon relaxation time can be described as:2
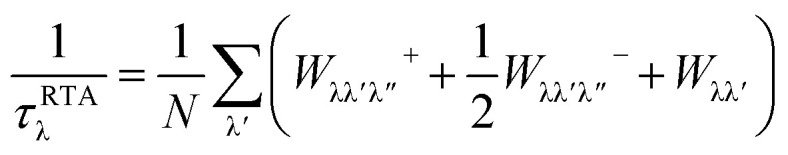
where *W*_λλ′_ is the isotope scattering rate and *W*_λλ′λ′′_^±^ are scattering rate of three-phonons absorption (+) and emission (−) processes, and modes λ, λ′ and λ′′ satisfy quasi-momentum conservation. *W*_λλ′λ′′_^±^ can be calculated from the Fermi's golden rule as follow:3
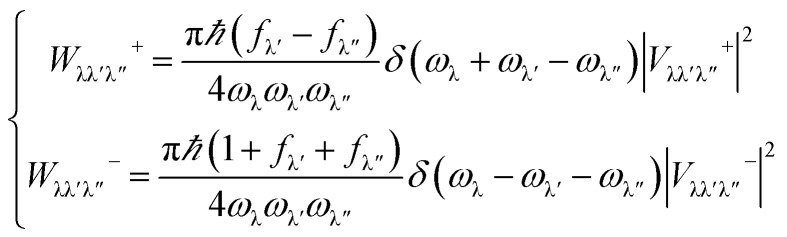
Here, *V*_λλ′λ′′_^±^ are scattering matrix elements depending on the third-order anharmonic force constants (3^rd^ IFCs).

The first principle calculation was performed based on density functional theory (DFT) as implemented in Vienna *Ab initio* Simulation Package (VASP)^34^ within the generalized gradient approximation (GGA) functional of the Perdew–Burke–Ernzerhof (PBE).^35^ The convergence criteria for energies was set to 10^−7^ eV and a Γ-centered *k*-mesh of 21 × 21 × 1 is used to sample the Brillouin Zone (BZ) of primitive unit cell. The structure was fully relaxed until interatomic force is less than 10^−5^ eV Å^−1^ with a cutoff energy of 550 eV. The Gaussian smearing of 0.2 eV was used to calculate the electronic occupation. To solve the phonon Boltzmann transport equation, ShengBTE code^36^ was used. Both the 2^nd^ and the 3^rd^ IFCs were calculated *via* finite displacement method. 7 × 7 × 1 supercells and 3 × 3 × 1 *k*-mesh were used to ensure the convergence of calculation. To ensure the convergence of thermal conductivity, we considered the interactions up to the 7^th^ nearest neighbors (corresponding to the cutoff distance of 0.68 nm) and chosen a 101 × 101 × 1 *q*-mesh for phonon–phonon scattering calculation. Furthermore, we also calculated Born effective charges and dielectric constants based on the density functional perturbation theory (DFPT) to correct the dynamical matrix by considering the long-range electrostatic interactions. Because van der Waals (vdW) diameter of As atom is less than that of B atom, we chose vdW diameter (0.426 nm) of B atom as the effective thickness of the monolayer h-BAs. In our calculation, the phonon scattering by isotopic doping is considered, with the natural isotope abundance of 19.78% ^10^B, 80.22% ^11^B, and 100% ^75^As.

## 3. Results and discussion

### 3.1. Phonon dispersion and weak π bond

**Fig. 1:**
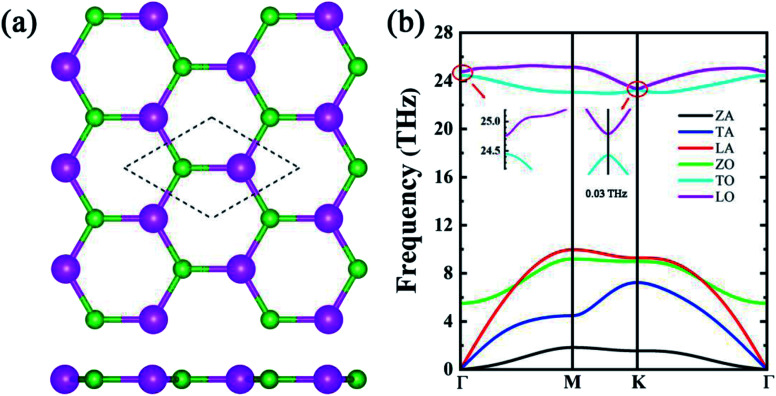
(a) Optimized structure and (b) phonon dispersion of h-BAs. The pink and green balls represent As and B atoms, respectively.

The fully optimized structure and corresponding phonon dispersion are illustrated in Fig. 1(a) and (b). The calculated lattice constant of 3.39 Å is coincident well with previous works.^18,24,25,33^ Considering the long-range electrostatic interactions, it is found that the LO (longitudinal optical)–TO (transverse optical) splitting appears at Brillouin Zone (BZ) center, which results in a slight frequency gap of 0.31 THz. This slight shift of LO branch stems from the similar electronegativity of B and As atom. The Born effective charge and dielectric constants are given in Table 1. The calculated Born effective charge for boron ion is *Z*(B)_xx_ = 1.744*e*, *Z*(B)_yy_ = 1.743*e*, for As ion is *Z*(As)_xx_ = −1.744*e*, *Z*(As)_yy_ = −1.743*e*, respectively, where *e* (positive) is elementary charge. The in-plane charge transfer between B and As atoms is comparable with that in other 2D materials, for example, InSe (2.33*e*), MoS_2_ (1.34*e*).^37^ On the other side, the charge transfer in the *z* direction is only 0.051*e*, much smaller than InSe (0.27*e*) and MoS_2_ (0.14*e*), because its ideal planar geometry. The main differences in phonon spectrum of h-BAs from most other planer hexagonal III–V compounds are the flat ZA branch and upward ZO branch. In h-BAs, the maximum frequency of ZA branch is only 1.81 THz, indicating ZA phonons exhibit slow group velocity and out-of-plane vibration is very weak. This result can be understood by the large atomic density of h-BAs (molar mass of h-BAs is 85.73 g mol^−1^, h-BN is 24.81 g mol^−1^) and weak B–As bonding along the out-of-plane direction. As a comparison, the IFCs of bonding atoms in graphene, h-BN and h-BAs are given in Table 2. Obviously, *Φ*_zz_ of B–As is much smaller than in-plane matrix elements and those of the other two materials. In planer honeycomb boron compounds with group-V elements, h-BAs has an unusual upward ZO branch, which results in a wide frequency-gap between ZA and ZO branch. Due to the important role of out-of-plane modes in thermal transport for planer structure, in the following sections, the effect of this frequency-gap on the three-phonon scattering processes will be discussed.

**Table 1:** Born effective charges (Z*) of B and As atoms and the dielectric constants (*ε*) of h-BAs

Component	Z*(B)	Z*(As)	*ε*
xx	1.744	−1.744	4.678
yy	1.743	−1.743	4.678
zz	−0.051	0.051	1.162

**Table 2:** IFCs of bonding atoms in graphene, h-BN and h-BAs in unit of eV Å^−2^. Here *x* corresponds to the longitudinal direction of bond

IFCs	C–C	B–N	B–As
*Φ*_xx_	25.21	21.72	12.70
*Φ*_yy_	10.47	8.05	2.65
*Φ*_zz_	6.11	5.03	0.54

### 3.2. Thermal conductivity and unusual frequency-independent region in phonon relaxation time

**Fig. 2:**
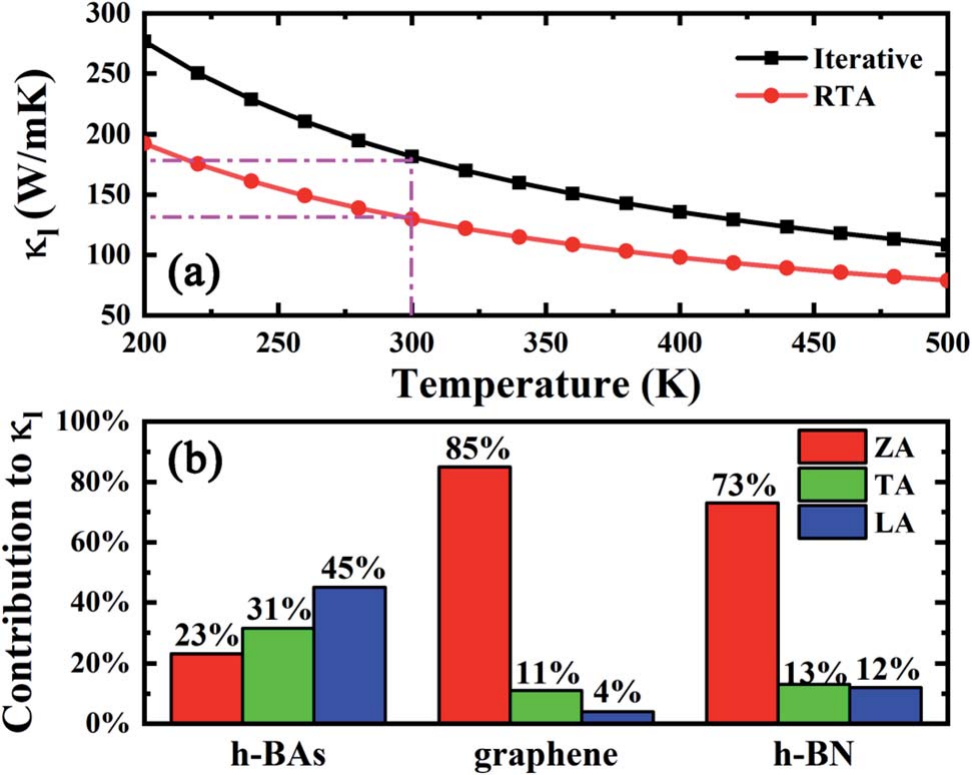
(a) Lattice thermal conductivity (*κ*_l_) of h-BAs as a function of temperature. (b) Percentage contribution to *κ*_l_ from each acoustic branch (ZA, TA and LA) in h-BAs, graphene and h-BN. The data about graphene and h-BN is from ref. 13.

The iterative solution of thermal conductivity as well as the one under relaxation time approximation (RTA) are illustrated in Fig. 2(a). At 300 K, the iterative solution of thermal conductivity is 181 W m^−1^ K^−1^, higher than many other narrow-gap semiconductors such as monolayer MoS_2_ (ref. 38) (87.6 W m^−1^ K^−1^), stanene^39^ (11.6 W m^−1^ K^−1^) and phosphorene^40^ (36 and 110 W m^−1^ K^−1^ along armchair and zigzag direction, respectively). We also investigate the isotope scattering effect on the thermal conductivity of h-BAs. The calculated results show that the thermal conductivity of pure h-BAs is only 3.7% higher than the natural one (19.78% ^10^B, 80.22% ^11^B, and 100% ^75^As), which is much lower than that in h-BN (∼40%).^41^ The weak isotopic effect in h-BAs can be explained by Matthiessen's rule. h-BAs has remarkably stronger phonon–phonon scattering strength than that in h-BN. The thermal conductivity of h-BAs is mainly determined by the phonon anharmonicity and isotope scattering effect has a minor influence on the thermal conductivity. In addition, the solution of thermal conductivity under RTA is about 72% of the iterative one, which is a common phenomenon because the normal (N) processes are treated resistive. As phonon hydrodynamics in h-BAs is weak, it is not our major focus in the present work. In the following, all the results and discussion are based on RTA calculation.

**Fig. 3:**
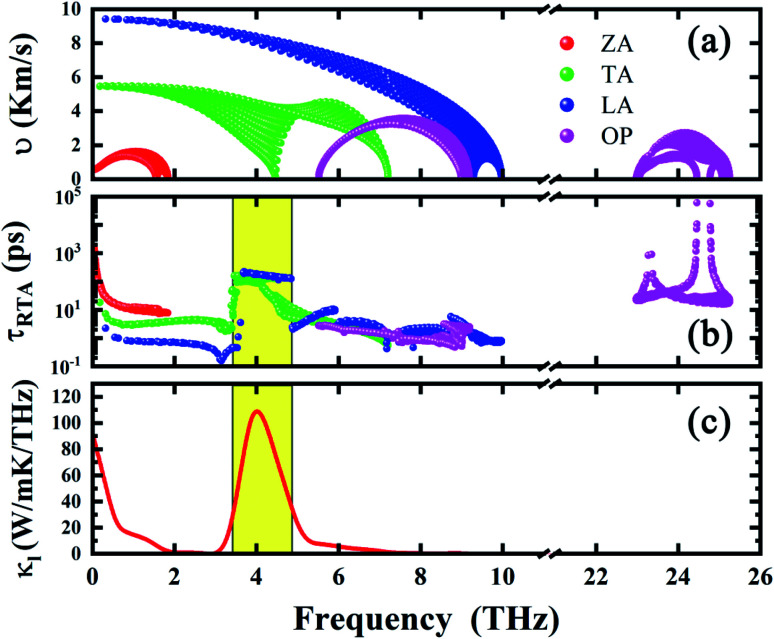
(a) Group velocity, (b) relaxation time and (c) thermal conductivity of h-BAs as a function of frequency. The frequency-independent relaxation time platform range is highlighted by yellow shadow.

Unlike other monolayer hexagonal structure such as graphene and h-BN in which ZA modes dominates the thermal conductivity due to the phonon scattering selection rule, h-BAs presents a relative low contribution from ZA modes (23%) as shown in Fig. 2(b). This is much lower than that of graphene (85%) and h-BN (73%).^42^ Meanwhile, IA (in-plane acoustic) modes play dominant role in thermal conductivity (76%) of h-BAs. To get the insight into the unusual phonon thermal transport property, we extract the phonon relaxation time and group velocity, as shown in Fig. 3(a) and (b). From the aspect of group velocity, it is found that LA (longitudinal acoustic) branch has the group velocity of 9.3 × 10^3^ m s^−1^ in long-wavelength limit. The remarkable gap of group velocity between LA an ZA phonons implies the in-plane (σ) bonding is much stronger than out-of-plane (π) bonding. This result is consistent with the analysis of interatomic force constants of B–As bond as given in Table 2. It is worth noting that although thermal conductivity of h-BAs is more than two times of that of monolayer MoS_2_, its group velocity is comparable with the longitudinal sound velocity of MoS_2_, which is around 7 × 10^3^ m s^−1^ measured experimentally.^43^ The relative slow group velocity in h-BAs cannot explain the abnormal high thermal conductivity. Therefore, next we focus on another critical factor, the phonon relaxation time.

Unexpectedly, we found a frequency-independent “platform region” in relaxation time of TA and LA modes. The “platform” for TA (LA) branch, occurs at about 3.47 (3.61) THz, and vanishes at 4.16 (4.86) THz, respectively. In the platform region, the magnitude of relaxation time increases up to 10^2^ ps. In Fig. 3(c), we present the frequency-resolved thermal conductivity (thermal conductivity *versus* frequency, namely, 
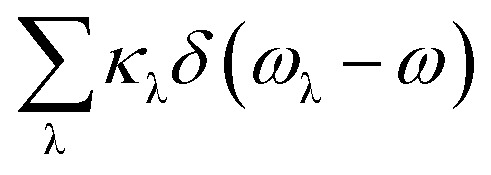
), which has two obvious peaks. The first one just covers low frequency range, indicating the major contribution of low frequency phonons. The second one has higher peak value and appears within the platform region, which demonstrate the unusually high contribution of corresponding IA modes to thermal conductivity.

To further understand the high thermal conductivity of h-BAs and explain the forming of platform, we consider the following scattering processes in low-frequency region: ZA + ZA → IA. As shown in Fig. 1(b), ZA branch of h-BAs is flat in low frequency region, the maximum frequency of ZA branch is only 1.81 THz. Therefore, considering the energy conservation requirement as *ω*_ZA1_ + *ω*_ZA2_ = *ω*_LA_ (*ω*_TA_), this phonon scattering process is greatly suppressed, in particular for LA or TA phonons with frequency higher than 3.62 THz. This is the underlying mechanism for the high relaxation time in the ‘platform’ region.

### 3.3. Comparative study with monolayer h-GaN

Although h-BAs has higher atomic density, its thermal conductivity is comparable with that of h-BN. Next, we compare it with another III–V semiconductor, h-GaN, because both of them have the similar atomic density. To make a comparative study, in the calculation of thermal conductivity of h-GaN, all the DFT calculation parameters, size of supercells and BTE calculation parameters are in line with those of h-BAs. The only difference is the effective thickness which is 0.37 nm in h-GaN.

**Fig. 4:**
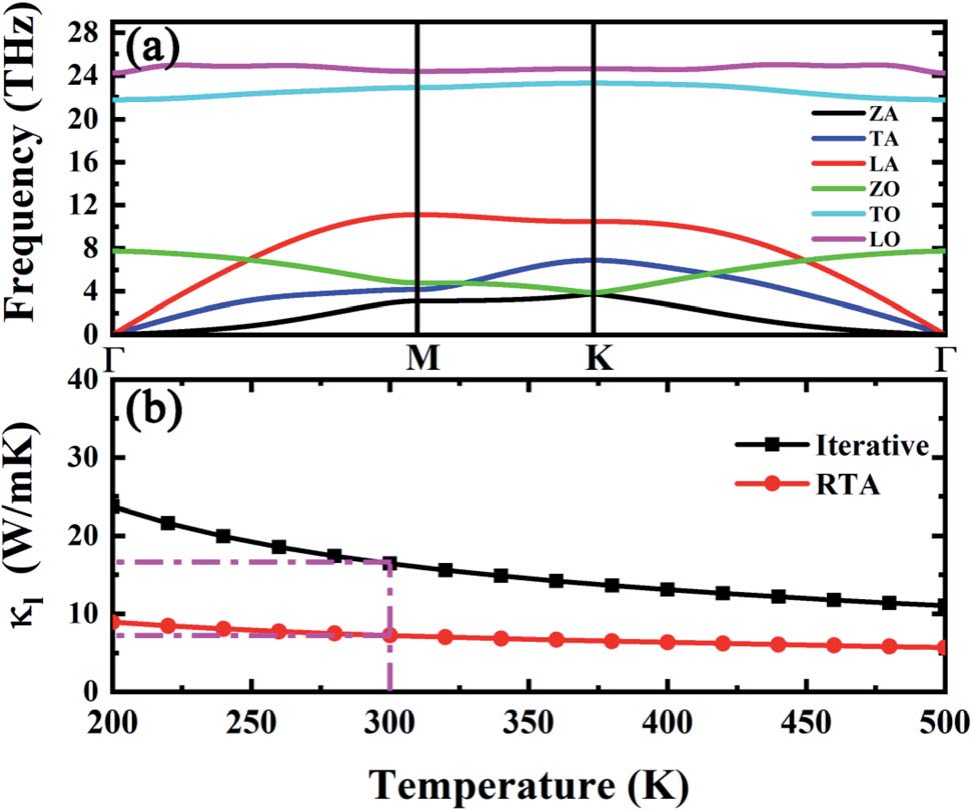
(a) Phonon dispersion and (b) thermal conductivity of h-GaN *versus* temperature.

The phonon dispersion of h-GaN is shown in Fig. 4(a). The LO–TO splitting leads to a 2 THz frequency shift at Γ point due to the large electronegativity difference between N and Ga atom. At 300 K, the iterative solution of thermal conductivity of h-GaN and the one from RTA is 16 and 7 W m^−1^ K^−1^ (see Fig. 4(b)), respectively.

**Fig. 5:**
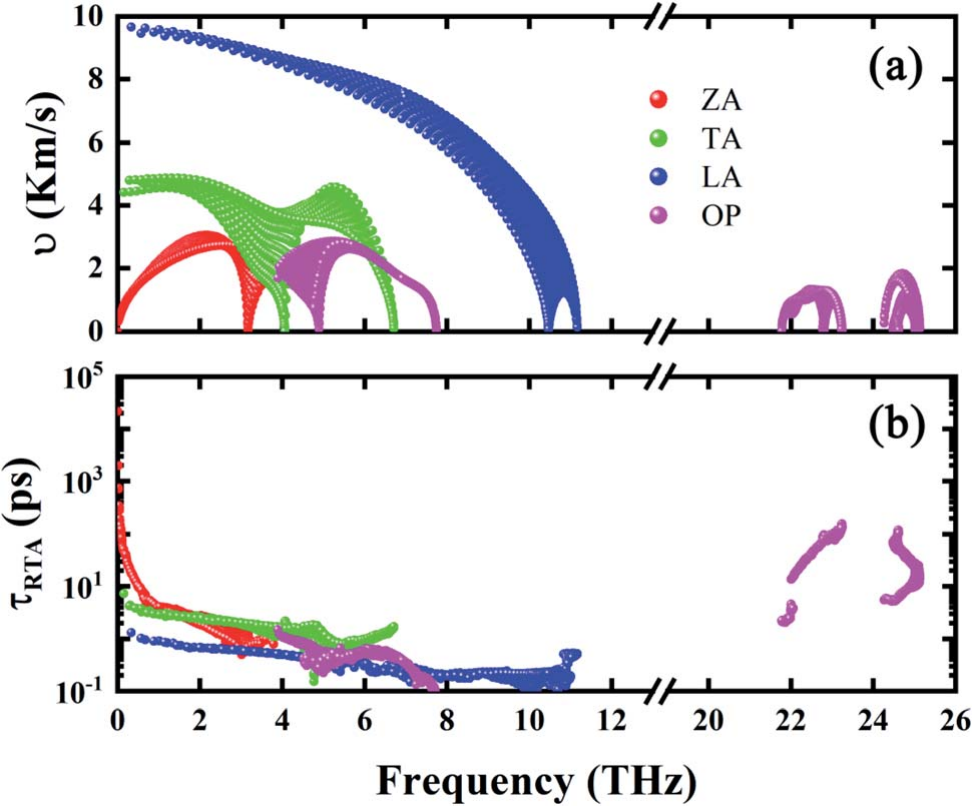
(a) Phonon group velocity and (b) relaxation time of h-GaN as a function of frequency.

**Fig. 6:**
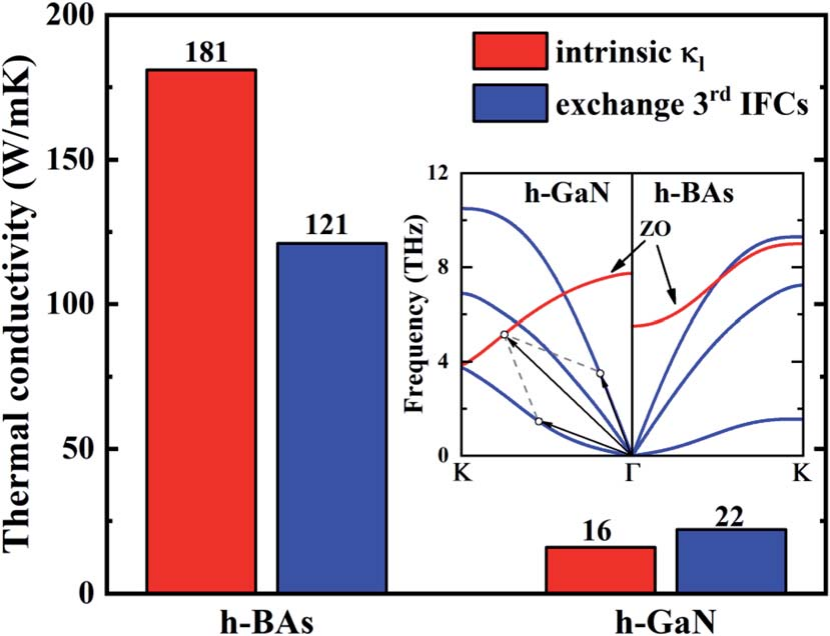
Intrinsic thermal conductivity and the one obtained after exchanging the 3^rd^ IFCs of h-BAs and h-GaN at 300 K. The inset is comparison of phonon dispersions between those two materials, indicating the opposite trends in ZO branch.

Comparing with h-BAs, 2D h-GaN has the same honeycomb planar structure, comparable average atomic mass and in-plane bonding stiffness, but much lower thermal conductivity. To explore the physical insights attribute to this abnormal phenomenon, we calculated the phonon group velocity and relaxation time of h-GaN. As shown in Fig. 5(a) and (b), it is shown that the difference in thermal conductivity between h-BAs and h-GaN mainly comes from their different phonon relaxation time. Except ZA branch, the relaxation times of other acoustic branches are less than 10 ps, which are one order of magnitude lower than that of IA branch in h-BAs. We also recalculated the thermal conductivity by only exchanging the 3^rd^ IFCs of each other. This operation can fix the phonon–phonon scattering channels and only change the scattering matrix elements *V*_λλ′λ′′_. This method has been used to provide physical insights of the thermal transport in fully filled skutterudite.^44^ By comparing the results before and after exchanging the 3^rd^ IFCs, we can clarify whether 2^nd^ IFCs or 3^rd^ IFCs plays the major role in the thermal conductivity. As shown in Fig. 6, by exchanging the 3^rd^ IFCs, the thermal conductivity of h-BAs is reduced by 33.2% and that of h-GaN is increased by 37.5%. This demonstrates that the 3^rd^ IFCs is not the major origin of difference in thermal conductivity between h-BAs and h-GaN.

Comparing the phonon dispersion of h-BAs and h-GaN in low frequency regions as shown in Fig. 6, we find unlike h-BAs, the ZO branch in h-GaN exhibits an obvious downward trend from the center to boundary of Brillouin zone, which provides more phase space for phonon scattering process such as ZA + IA → ZO. This difference greatly increases the phonon scattering channels and reduces relaxation time of in-plane acoustic phonon modes in h-GaN.

## 4. Summary

In summary, by solving BTE based on first principle calculation, we revealed the abnormal diffusive thermal transport behaviors in h-BAs and compared the thermal conductivity of h-BAs with h-GaN. At 300 K, the thermal conductivity of h-BAs is up to 181 W m^−1^ K^−1^, obviously larger than that of h-GaN with 16 W m^−1^ K^−1^. This abnormal high thermal conductivity originates from the unexpected platform region with long phonon relaxation time of IA modes. Based on the selection rule, we found this platform in phonon relaxation time stems from suppressed ZA + ZA → IA scattering channel. Moreover, IA modes make major contribution to thermal conductivity, different from that of graphene and h-BN. This is not only because the IA modes have long relaxation time and high group velocity, but also the flat ZA branch due to weak π-bond strength. Comparing the phonon dispersion of h-BAs with h-GaN, the main difference is that ZO branch of h-GaN has an obvious downward trend from the BZ center to boundary, which increases the phase space of ZA + IA → ZO scattering, leading to the low thermal conductivity.

## Conflicts of interest

There are no conflicts to declare.

## Supplementary Material
